# Quantum Dot Doping-Induced Photoluminescence for Facile, Label-Free, and Sensitive Pyrophosphatase Activity Assay and Inhibitor Screening

**DOI:** 10.3390/nano9010111

**Published:** 2019-01-18

**Authors:** Yishen Tian, Lijie Hao, Chao Wang, Xiaoyan Yang, Shufeng Liu

**Affiliations:** Key Laboratory of Optic-Electric Sensing and Analytical Chemistry for Life Science, Ministry of Education, College of Chemistry and Molecular Engineering, Qingdao University of Science and Technology, Qingdao 266042, China; tianysqd@163.com (Y.T.); haolijiequst@163.com (L.H.); chwang2018@yeah.net (C.W.); yangxiaoyan_zh@126.com (X.Y.)

**Keywords:** quantum dot doping, pyrophosphatase activity, fluorescence detection, pyrophosphate, copper ion

## Abstract

Development of simple, convenient, and sensitive assay methods for pyrophosphatase (PPase) activity is of importance, for disease diagnosis and drug discovery. Herein, a simple, rapid, label-free, and sensitive fluorescence sensor for PPase activity assay is developed, using Cu^2+^ doping-induced quantum dot (QD) photoluminescence as a signal reporter. The Cu^2+^ doping of ZnSe QD can induce a dopant-dependent emission response, which will be inhibited after the premixing of Cu^2+^ with pyrophosphate (PPi), to form a Cu^2+^-PPi complex. Then, the hydrolysis of PPi into phosphate (Pi), specifically catalyzed by PPase, liberates the free Cu^2+^ to regain the QD doping for the fluorescence response, which is highly dependent on the PPase activity. The PPase can be sensitively and selectively assayed, with a detection limit of 0.1 mU/mL. The developed sensing strategy can be also employed for the PPase inhibitor screening. Thus, the current QD doping-based sensing strategy offers an efficient and promising avenue for Cu^2+^, PPi, or PPase-related target analysis, and might hold great potential for the further applications in the clinical disease diagnosis.

## 1. Introduction

Inorganic pyrophosphatase (PPase), as a ubiquitous hydrolytic enzyme in biological systems, can specifically catalyze the hydrolysis of pyrophosphate (PPi) into orthophosphate (Pi). Such a hydrolysis process is always accompanied by energy release, thus, accommodating the thermodynamic impetus for many biosynthetic reactions [1,2,3]. It, thus, demonstrates a very critical role in a series of important biological processes, for example, carbohydrate and lipid metabolism, DNA synthesis, and other biochemical transformations [4,5,6]. The abnormal level of PPase has been directly connected with several clinical diseases, including hyperthyroidism, colorectal cancer, and lung adenocarcinomas [7,8,9]. It has also served as an important therapeutic target for drug development. The identification of PPase activity is, therefore, of paramount importance for understanding relevant physiological and pathological processes, and also for some disease diagnosis and clinical medicine.

Until now, many methods including radiochemical, enzymatic, and optical, etc. have been proposed for the PPase activity assay [10,11,12]. The optical sensing method is especially attractive, owing to its distinctive advantages, such as simplicity, speed, homogenous, and high sensitivity. The previously reported optical methods for PPase assay could be simply classified into nanomaterials or organic dyes-based types, both of which are usually mediated by Cu^2+^/PPi, to achieve the signal switching. Examples of nanomaterials-based strategy include the design of cysteine-stabilized Au nanoparticles [13], 11-mercaptoundecanoic acid-capped Au nanoclusters [14], gold-silver bimetallic nanoclusters [15], etc. as colorimetric or fluorescent nanomaterials for determination of the PPase activity. The organic dye-based PPase activity assays were reported, based on click chemistry [16], Cu^2+^-regulated o-phenylenediamine oxidation [17], or the synthesized organic probes for the direct discrimination between phosphate and pyrophosphate [18]. These optical strategies have achieved the effective detection of PPase, however, the synthesis, modification, and even purification operation of organic probes or nanoprobes involved in most of these methods increases the assay complexity, restricting their wide applications to some extent. Thus, development of simple, convenient, cost-effective, and sensitive methods for profiling PPase activity is still highly demanded to accommodate better for PPase-related disease diagnosis and drug discovery.

Quantum dot (QD) can now be regarded as one of the most attractive nanomaterials used for biosensor fabrication, owing to its easy to synthesis, excellent photoluminescence ability, good stability, and biocompatibility [19,20,21,22]. However, surface modification or even complex bioconjugation steps are usually required, before its exploitation for various applications. The QD doping with various metal ions has been largely reported to alter the bandgap energy and induce a dopant-specific emission response [23,24,25]. It, thus, might provide a convenient and promising pathway for monitoring or analysis of specific metal ion or even metal ion-related targets. Nevertheless, the attempt of such QD doping principle for biosensor fabrication is still rarely reported. Liu et al. developed an on–off–on luminescent pyrophosphate probe, based on the use of Mn-doped ZnS quantum dots [26]. The QD doping by Hg^2+^ has been also explored for DNA and protein detection, which could be considered as the typical examples for the application of QD doping in biosensor fabrication [27,28].

**Figure 1:**
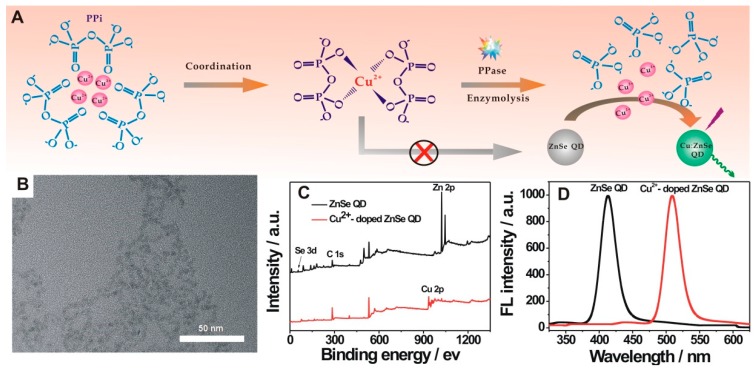
(**A**) Schematic illustration of pyrophosphatase (PPase) sensing strategy; (**B**) Transmission electron microscopy (TEM) characterization of the prepared ZnSe quantum dot (QD); (**C**) X-ray photoelectron spectroscopy (XPS) spectra for the ZnSe QD in the absence (black line) or presence (red line) of excess Cu^2+^; (**D**) Fluorescence spectra for the ZnSe QD (black line), and doped QD with 100 μM Cu^2+^ (red line).

Herein, a proof-of-concept for simple and powerful PPase sensing is described with the use of Cu^2+^ doping-induced ZnSe QD photoluminescence, as a new signal reporting element. The schematic illustration for PPase sensing is shown in Figure 1A. First, it was found that the Cu^2+^ doping of ZnSe QD could yield a dopant-dependent emission response, at 510 nm. However, such an emission could be inhibited after the coordination of Cu^2+^ with PPi to form a Cu^2+^-PPi complex [13,29]. Upon addition of PPase, the PPi was then specifically hydrolyzed into Pi, to release the chelated Cu^2+^, which in turn regained the QD doping for the emission response related with PPase determination. The fluorescence intensity of the resultant solution was highly dependent on the concentration of free Cu^2+^, thus, could indirectly indicate the level of PPase activity. Additionally, the developed system could be applied for PPase inhibitor screening.

## 2. Materials and Methods

### 2.1. Materials and Chemicals

Baker’s yeast inorganic PPase (EC3.6.1.1), L-glutathione (GSH, 98%), selenium powder (99.99%), glucose oxidase (GOx), lysozyme, sodium borohydride (98.0%), and tris(hydroxymethyl)aminomethane were purchased from Sigma-Aldrich (St. Louis, MO, USA). One unit of Baker’s yeast PPase can liberate 1.0 μmol of inorganic orthophosphate/min at pH 7.2 and 25 °C, referring to Sigma’s unit definition. Exonuclease I (Exo I) and Exonuclease III (Exo III) were purchased from the New England Biolabs Company (Beverly, MA, USA). CuSO_4_·5H_2_O, sodium pyrophosphate and NaF were purchased from the Aladdin Industrial Corporation (Shanghai, China). Fetal calf serum was obtained from Sangon Biotech. Co., Ltd. (Shanghai, China). All other reagents were analytically purity graded and used as received, without further purification.

### 2.2. Preparation of ZnSe QD

ZnSe QD was prepared, based on the previously reported procedures; listed below [27,28]. The NaHSe precursor solution (250 mM) was first obtained by adding selenium powder (0.0197 g) and sodium borohydride (0.02 g), into 1 mL of ultrapure water, at room temperature. Then, the NaHSe solution (25 μL, 250 mM) was rapidly mixed with zinc acetate (125 μL, 100 mM), GSH (93.75 μL, 200 mM), and NH_4_HCO_3_ solution (281.25 μL, 0.2 M, pH 12.3). After vortex agitation for 10 s, the resulting ZnSe QD was obtained. Followed by centrifugation (13,500 rpm, 10 min) via a centrifugal device (3K, Pall Corporation, Ann Arbor, MI, USA), the ZnSe QD was recovered and diluted to the original volume with NH_4_HCO_3_ solution (0.2 M, pH 12.3).

### 2.3. Fluorescent Assay for PPase

To study the Cu^2+^ doping effect on the ZnSe QD photoluminescence, 50 μL of varied concentrations of Cu^2+^ were mixed with the freshly prepared ZnSe QD (100 μL), tris-HCl buffer (700 μL, 50 mM, pH 7.5), and H_2_O (150 μL), and then conducted for fluorescence measurement.

To explore the effect of PPi on the fluorescence responses of QD, 50 μL of different concentrations of PPi were first mixed with Cu^2+^ solution (50 μL, 200 μM), and then the mixtures were added into the freshly prepared ZnSe QD (100 μL), tris-HCl buffer (700 μL, 50 mM, pH 7.5), and H_2_O (100 μL), for fluorescence measurement.

The PPase activity assay was performed, according to the following procedures. First, 50 μL of freshly prepared PPase solution, with different activities, were added into the mixture solution containing Mg^2+^ (20 µL, 1 mM), Cu^2+^ (50 μL, 200 μM), PPi (50 μL, 400 μM), tris-HCl buffer (700 μL, 50 mM, pH 7.5), and H_2_O (30 μL). The above solution was incubated at 37 °C for 60 min. Then, the PPase-treated solutions were mixed with the ZnSe QD solution (100 μL), for fluorescence measurement.

### 2.4. PPase inhibition Assay

First, 30 μL of varied concentrations of NaF were mixed with the freshly prepared PPase solution (50 μL, 200 mU/mL). Then, these NaF-treated PPase solutions were added into the mixture solution containing Mg^2+^ (20 µL, 1 mM), Cu^2+^ (50 μL, 200 μM), PPi (50 μL, 400 μM), and tris-HCl buffer (700 μL, 50 mM, pH 7.5). After the incubation at 37 °C for 60 min, the resulting mixtures were used for fluorescence measurement.

### 2.5. Instruments

Fluorescence spectra were recorded on a F-2700 spectrometer (Hitachi, Tokyo, Japan), with the set parameters (scan rate, 1500 nm/min; excitation wavelength, 350 nm; 24 photomultiplier voltage, 700 V; slits for excitation and emission, 10 nm/10 nm). Transmission electron microscopy (TEM) characterization was performed on a JEM-2100F field emission electron microscope (JEOL, Tokyo, Japan), at an accelerating voltage of 200 kV. X-ray photoelectron spectroscopy (XPS) measurements were conducted on an ESCALAB 250Xi (Thermo Fisher, Waltham, MA, USA) spectrometer with Al Ka excitation (1486 eV).

## 3. Results

### 3.1. QD Photoluminescence Regulated by Cu^2+^/PPi

ZnSe QD was obtained with the use of Zn(OAc)_2_ and NaHSe as precursors and glutathione, as ligand. As characterized by TEM, the ZnSe QD showed a quasi-spherical shape with a mean diameter of about 3.3 nm (Figure 1B). The XPS spectra for the ZnSe QD, in the presence of Cu^2+^, or not, were shown in Figure 1C. Upon incubation of ZnSe QD with Cu^2+^, the peak for Cu 2p could be clearly observed, besides the typical peaks for ZnSe QD (Se 3d, C 1s, Zn 2p), suggesting the Cu^2+^ incorporation into the ZnSe QD. The fluorescence responses of ZnSe QD doped by Cu^2+^, or not, were shown in Figure 1D. Only an emission response at around 412 nm could be observed for the initial ZnSe QD. After Cu^2+^ doping, such an emission response vanished but a new peak, centered at 510 nm, appeared, revealing the Cu^2+^ doping-induced QD photoluminescence. Although the corresponding mechanism for QD doping needs to be further explored, it could be still regarded as an excellent signal generating and reporting element to probe its potential applications.

**Figure 2:**
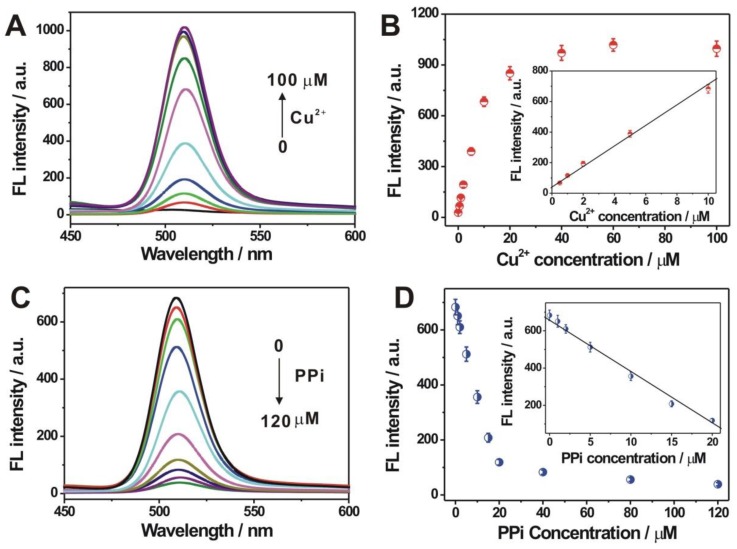
(**A**) Fluorescence spectra of ZnSe QD doped with varied concentrations of Cu^2+^ (0–100 µM); (**B**) The calibration curve of the fluorescence intensity at 510 nm versus Cu^2+^ concentration. The inset shows the linear curve between fluorescence intensity and Cu^2+^ concentration; (**C**) Fluorescence spectra of the ZnSe QD by adding the mixture containing 10 μM Cu^2+^ and varied concentrations of pyrophosphate (PPi) (0–120 μM); (**D**) The calibration curve between fluorescence intensity at 510 nm and PPi concentration. The inset shows the linear curve between fluorescence intensity and PPi concentration. Error bars in (**B**,**D**) for each data point, indicate the standard deviations, which were calculated on the basis of three repetitive experiments.

Furthermore, we explored the fluorescence responses of QD at various Cu^2+^ concentrations. It could be seen from Figure 2A that the fluorescence intensity increased gradually with the increasing Cu^2+^ concentration from 0 to 100 μM, indicating a high dependency of the fluorescence response of the doped-QD, on the Cu^2+^ concentration. Figure 2B shows the corresponding calibration curve. A linear plot could be achieved between the fluorescence intensity and the Cu^2+^ concentration (0.5–10 µM), with a correlation coefficient of 0.9947. In the following experiments, 10 μM of Cu^2+^ was adopted for the PPi and PPase assay. The effect of PPi on the fluorescence intensity of the QD was probed, by first mixing the PPi (0–120 μM) with 10 μM Cu^2+^, and then adding into the freshly prepared ZnSe QD. The fluorescence intensity of the QD decreased with the increase of the PPi concentration (Figure 2C). It could be explained that the complex formation between the Cu^2+^ and the added PPi, inhibited the Cu^2+^ doping for the fluorescence response decrease. The corresponding calibration curve of the fluorescence intensity versus the PPi concentration is shown in Figure 2D. The fluorescence intensity of the QD at 510 nm, decreased sharply with the increase of the PPi concentration from 0 to 20 μM, and reached a plateau after 20 μM. Additionally, a good linear plot for the fluorescence intensity with the PPi concentration (0–20 μM) could be obtained. In the current sensing system, if too much PPi is used, the PPase will hydrolyze the free PPi first, which will not be beneficial for the subsequent PPase activity assay. Thus, the PPi concentration of 20 μM was chosen in the following experiments.

### 3.2. Optimization of the Experimental Conditions

**Figure 3:**
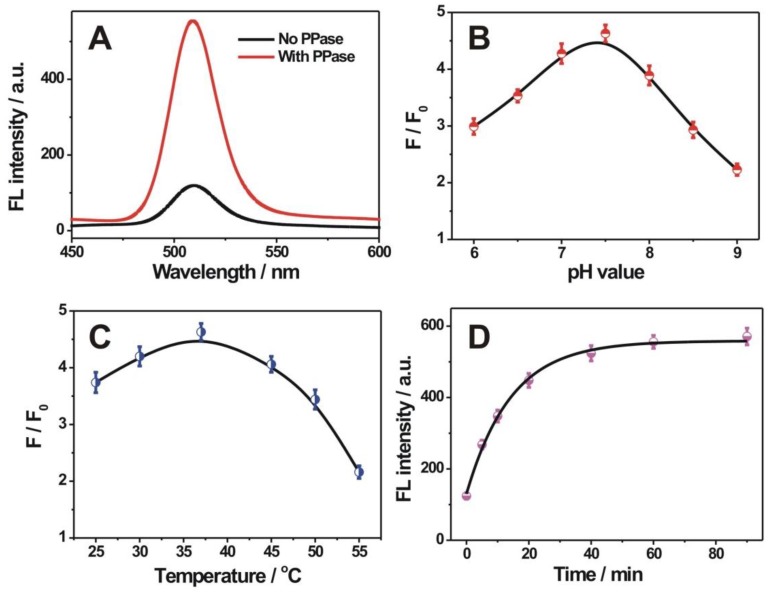
(**A**) Fluorescence spectra of the ZnSe QD mixture with Cu^2+^ + PPi (black line), and Cu^2+^ + PPi + PPase (red line); (**B**) Effect of the pH value on the fluorescence response toward PPase; (**C**) Effect of temperature on the fluorescence response toward PPase; (**D**) Effect of reaction time for the PPase-regulated hydrolytic process. The employed concentrations for Cu^2+^, PPi, and PPase were 10 µM, 20 µM, and 10 mU/mL, respectively. The *F* and *F*_0_ in Figure 3B,C represented the fluorescence responses obtained in the presence of 10 and 0 mU/mL PPase, respectively. Error bars in (**B**–**D**), for each data point, indicate the standard deviations, which were calculated on the basis of three repetitive experiments.

To verify the detection feasibility of the current sensing strategy toward PPase activity, the corresponding fluorescence spectra, in the presence and absence of PPase, were shown in Figure 3A. The addition of PPase into the premixed Cu^2+^ and PPi solution could induce a distinct increase of the fluorescence intensity of QD, at 510 nm, compared with that of no PPase, indicating that the catalytic hydrolysis of PPi into Pi by PPase, liberated the Cu^2+^ to regain the QD doping-induced photoluminescence. To achieve the best sensing capability toward the PPase activity, the other experimental conditions, including pH value, reaction temperature, and hydrolysis time, were also optimized. It could be seen from Figure 3B that a maximum signal-to-background ratio could be acquired at a pH 7.5 tris-HCl buffer solution. The reaction temperature would have an effect on the enzymatic process of PPase. It could be seen from Figure 3C that the 37 °C is the suitable temperature for maintaining the PPase activity, and could achieve better performance toward PPase than other tested temperatures. The hydrolysis time between PPi and PPase was also studied (Figure 3D). The fluorescence intensity of QD at 510 nm, increased with the increase of hydrolysis time and almost reached a plateau value, at 60 min (Figure 3D). Thus, a 60 min hydrolysis time was employed in the following PPase assay.

### 3.3. Sensing Performance toward PPase Activity

**Figure 4:**
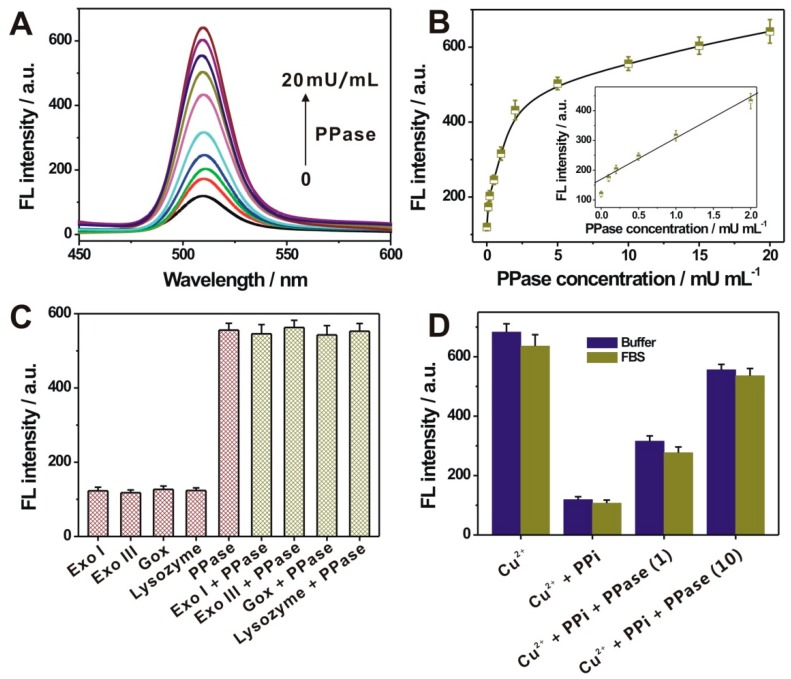
(**A**) Fluorescence spectra of QD upon incubation of different concentrations of PPase from 0 to 20 mU/mL; (**B**) Relationship of the fluorescence intensity of QD at 510 nm, with the PPase concentration. Inset shows the corresponding linear range. (**C**) The specificity of the proposed sensing strategy toward PPase, against Exo I, Exo III, GOx, and lysozyme. The concentration of PPase was 10 mU/mL, and the concentrations for all other proteins were 0.1 U/mL. (**D**) The fluorescence intensities of QD, into the mixture of Cu^2+^ (10 µM), Cu^2+^ (10 µM) + PPi (20 µM), and Cu^2+^ (10 µM) + PPi (20 µM) + PPase (1, 10 mU/mL), respectively, in the buffer solution and 5% diluted fetal bovine serum (FBS). Error bars in (**B**–**D**), for each data point, indicate the standard deviations, which were calculated on the basis of three repetitive experiments.

Under the optimized experimental conditions, the PPase activity was tested by the current sensing system. The fluorescence spectra recorded at different PPase concentrations were shown in Figure 4a. A stepwise increase of fluorescence intensity of QD could be observed upon increasing PPase concentrations from 0 to 20 mU/mL, suggesting a PPase concentration-controlled or dependent response manner. Figure 4B shows the fluorescence intensity of QD as a function of the PPase concentration. A good linear relationship of the fluorescence intensity with the PPase concentration, ranged from 0.1 to 2 mU/mL, could be obtained with a regression equation of *Y* (fluorescence intensity) = 169 + 138*X* (PPase concentration) and a correlation coefficient of 0.9926. The detection limit toward the PPase was achieved as 0.1 mU/mL, which was compared with the reported methods (Table 1). The selectivity for the PPase activity of the developed method was also investigated by using other non-specific proteins, including lysozyme, glucose oxidase (GOx), exonuclease I (Exo I), and exonuclease III (Exo III). As shown in Figure 4C, a remarkable fluorescence response could be only observed in the presence of PPase, and also, these control proteins could not interfere with the detection of PPase activity. To further evaluate the potential applications of the currently developed sensor, we challenged the detection toward Cu^2+^, PPi, and PPase spiked in a relatively complex biological matrix (5% diluted fetal bovine serum). The fluorescence responses toward these different species in the diluted serum were all comparable with that in buffer (Figure 4D), suggesting the applicative potential in the relatively complex biological samples.

### 3.4. PPase Inhibitor Screening

**Figure 5:**
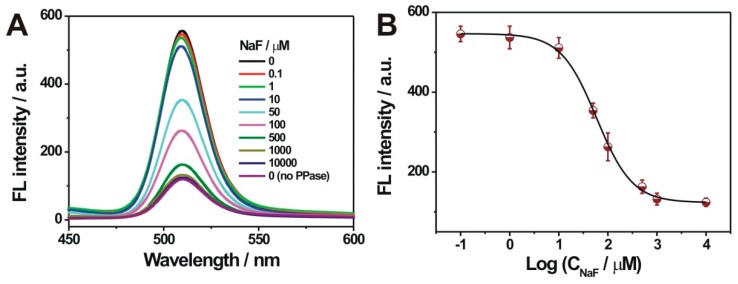
(**A**) Fluorescent spectra for different amounts of NaF treated PPase (10 mU/mL), into the mixtures containing Cu^2+^ (10 μM) and PPi (20 μM) in tris-HCl buffer (50 mM, pH 7.5); (**B**) The calibration curve of the fluorescence intensity with the logarithmic value of NaF concentration. Error bars indicate the standard deviations, which were calculated on the basis of three repetitive experiments.

The developed sensing system was also extended for the PPase inhibition evaluation, by using NaF as a typical PPase inhibitor. As shown in Figure 5A, the fluorescence intensity of the QD decreased stepwise, with the concentration increase of added NaF (0–10 mM). Such a trend was especially evident when the NaF concentration was over 10 µM. This strongly indicated the inhibition effect of NaF on the PPase activity. The calibration curve (fluorescence intensity versus the logarithm value of the NaF concentration) showed a typical sigmoidal profile (Figure 5B). The IC50 value (the inhibitor concentration that can cause 50% inhibition of the enzyme activity) was calculated to be about 58.07 µM, which was basically in accordance with the reported PPase activity assays [13,17]. Thus, the developed sensing system might be used for the screening of potential PPase inhibitors.

## 4. Conclusions

In conclusion, a simple, rapid, cost-effective, and sensitive strategy for PPase activity determination was demonstrated for the first time, using quantum dot doping-induced photoluminescence. The Cu^2+^ doping of ZnSe QD was first revealed to yield a dopant-specific emission peak. Then, the PPi—as an intermediate species—served for the Cu^2+^ constraint, through a strong coordination effect; and its specific hydrolysis into orthophosphate, by the PPase—for the Cu^2+^ liberation—in turn, regained the QD doping for the fluorescence response related with the PPase activity. The PPase could be assayed sensitively and selectively with a detection limit of 0.1 mU/mL. The current QD doping-based sensing strategy exhibited several advantages, such as high sensitivity and selectivity, the simple mix-and-detect operation in needless of washing and separation steps, and rapidness (within 60 min). Additionally, it avoided any labelling or modification operation commonly encountered in most QD-based sensing strategy. It could also be efficiently applied for the PPase inhibitors screening. Thus, it opens a convenient and promising avenue for the Cu^2+^-related target detection and might hold a great potential for the further applications in the clinical diagnosis of Cu^2+^, PPi, or PPase-related diseases.

## Figures and Tables

**Table 1:** Detection performance comparison toward PPase activity by different methods.

Method	Detection Limit (mU/mL)	Linear Range (mU/mL)	Strategy	References
Colorimetry	10	25.0–400.0	Gold nanoparticles	[13]
Fluorescence	0.03	0.1–30.0	Au-Ag NCs	[15]
Fluorescence	0.2	0.2–50.0	o-Phenylenediamine oxidation	[17]
Fluorescence	1.0	1.0–200.0	Graphene quantum dots	[30]
Fluorescence	1.3	3.0–40.0	Copper nanoclusters	[31]
Eletrochemistry	0.6	1.0–50.0	G-quadruplex-Cu^2+^ DNAzyme	[32]
Fluorescence	0.1	0.1–2.0	Quantum dot doping	This work
